# Cell Adhesion Molecules in Schizophrenia Patients with Metabolic Syndrome

**DOI:** 10.3390/metabo13030376

**Published:** 2023-03-02

**Authors:** Anastasiia S. Boiko, Irina A. Mednova, Elena G. Kornetova, Arkadiy V. Semke, Nikolay A. Bokhan, Svetlana A. Ivanova

**Affiliations:** 1 Mental Health Research Institute, Tomsk National Research Medical Center, Russian Academy of Sciences, Aleutskaya, Str. 4, 634014 Tomsk, Russia; 2 Department of Psychiatry, Addictology and Psychotherapy, Siberian State Medical University, Moskovsky Trakt, 2, 634050 Tomsk, Russia; 3 Department of Psychotherapy and Psychological Counseling, National Research Tomsk State University, 634050 Tomsk, Russia

**Keywords:** schizophrenia, metabolic syndrome, BMI, cell adhesion molecule, sICAM, sVCAM, sNCAM

## Abstract

Metabolic syndrome (MetS) is a common comorbidity of schizophrenia and significantly shortens life expectancy of the patients. Intercellular (ICAM), vascular (VCAM), and neural (NCAM) cell adhesion molecules (CAMs) mediate neuroinflammatory processes, and their soluble forms (e.g., sICAM) in plasma are present in parallel with their cell-bound forms. In this study, their serum levels were examined in 211 white Siberian patients with paranoid schizophrenia (82 patients with and 129 without MetS according to the 2005 International Diabetes Federation criteria). Serum levels of CAMs were determined with Magpix and Luminex 200 (Luminex, Austin, TX, USA) using xMAP Technology. The level of sICAM-1 was significantly higher and that of sVCAM-1 significantly lower in patients with MetS compared to patients without MetS. Levels of NCAM did not differ between the groups. More pronounced Spearman’s correlations between CAMs, age, duration of schizophrenia, and body–mass index were observed among patients without MetS than among patients with MetS. Our results are consistent with MetS’s being associated with endothelial dysfunction along with other components of inflammation. Through these endothelial components of peripheral inflammatory processes, MetS might induce intracerebral neuroinflammatory changes, but further investigation is needed to confirm this.

## 1. Introduction

Metabolic syndrome (MetS) and obesity are widespread among patients with schizophrenia; according to different authors, the prevalence of MetS among these pa-tients during antipsychotic treatment is in the range from 28% to 46% [1,2,3], and that of obesity from 16.4% to 48.9% [4,5]. Many factors common between mentally healthy in-dividuals and patients with schizophrenia contribute to the development of MetS, and the key ones are a sedentary lifestyle and an unbalanced diet [6]. At the same time, in patients with schizophrenia, MetS is most often considered a side effect of antipsy-chotic therapy: on haloperidol, metabolic disorders develop in 39.4% of the patients; on clozapine, in 44.7%; on amisulpride, in 33.3%; on olanzapine, in 34%; on quetiapine, in 18.8%; and on risperidone, in 35% of the patients [7].

MetS is a predisposing factor for cardiovascular pathologies and is regarded as a mortality risk factor in patients with mental disorders. Information on the pathogenesis of MetS can be obtained by studying hormones regulating metabolism and their genes, which in turn can also be considered potential biomarkers of the susceptibility to MetS and to lipid metabolism disorders [8,9]. Currently, aside from the generally accepted signs of MetS (central obesity, hyperglycemia, dyslipidemia, and blood pressure elevation), an important role is attributed to other and newer signs of MetS, such as microalbuminuria, cytokines, prothrombotic and fibrinolytic factors, and oxidative stress [10,11,12,13].

Molecular neurobiological research suggests that inflammation and immune activation may affect the functioning and plasticity of neurons and can switch microglial cells to a proinflammatory state associated with neurodegeneration [14,15,16]. Cell adhesion molecules (CAMs) are important for the formation and maintenance of neural structures [17], whereas disturbances of neural structures are observed in mental disorders [18,19,20]. A number of intercellular immunoglobulin-like transmembrane glycoproteins are expressed in chronic inflammatory conditions [21,22]. 

Intercellular adhesion molecule 1 (ICAM-1) is a transmembrane adhesion protein that participates in the migration of leukocytes to a site of inflammation [23]. Its functions are diverse and not completely clear [24], but there is evidence that ICAM-1 can play a modulating role in inflammation [25]. The expression of sICAM-1 is increased in various pathological conditions, for example, bacterial sepsis [26], type II diabetes mellitus [27], preeclampsia [28], and atherosclerosis [29]. ICAM-1 is expressed in the central nervous system (CNS) in human forebrain white and gray matter endothelial cells and astrocytes and in microglial cells [21]. Investigation of ICAM-1 in psychiatric disorders is relevant for two reasons: this protein plays a key part in the blood-brain barrier (BBB), is important for the pathogenesis of schizophrenia and other psychiatric disorders, and is a marker of inflammation. Some authors report an increase in ICAM-1 expression in schizophrenia and depressive disorders, including bipolar affective disorder [16,21]. In human obesity, overexpression of the soluble form (sICAM-1) positively correlates with the amount of abdominal fat [30]. Some studies have shown that sICAM-1 levels are also elevated in obesity and correlate with central obesity [29,30,31,32]. 

Vascular cell adhesion molecule 1 (VCAM-1) is a membrane-bound receptor of adhesion molecules and a member of the immunoglobulin family. The soluble form (sVCAM-1) is considered a ligand for leukocyte integrins, which mediate binding and transformation of monocytes and lymphocytes as well as strong attachment and transendothelial migration of leukocytes [33]. Endothelial cells are not only a passive barrier but also immunologically active themselves. They can produce chemokines, and endothelial sVCAM-1 is associated with age- and inflammation-induced activation of microglia, impaired neurogenesis, and cognitive deficits [34,35]. Several stimuli, including cytokines, lipoproteins, products of glycation, hypertension, and oxidants can enhance VCAM-1 expression [33]. Activated endothelial cells initially upregulate selectins (e.g., P-selectin) to slow leukocytes on the endothelial surface before firm adhesion via integrins and immunoglobulin superfamily CAMs (e.g., ICAMs and VCAMs). On leukocytes, the expression of integrin receptors, which may bind CAMs and facilitate transmigration of these cells across the BBB, has been shown to be increased in schizophrenia [16].

Neural cell adhesion molecule (NCAM) is another member of the immunoglobulin superfamily [36]. NCAM plays a crucial role in intercellular adhesion, neuronal migration, synaptic plasticity, and brain development and is involved in learning and memory formation processes [37,38,39]. NCAM is primarily expressed in neurons and not only participates in intercellular homotypic adhesion but also functions as a signaling receptor. NCAM enables endothelial cell adhesion and communication with other cells, such as pericytes and astrocytes. NCAM expression has been detected both in neurons and in myocytes in the zone of neuromuscular contact, on lymphocytes, and in organs of the vascular and other systems. These CAMs may be shed from the cell surface by different mechanisms, and their soluble form is released into the circulation. Soluble forms of NCAM are modulators of NCAM-mediated cell behavior. Given that NCAM plays an important part in the development of the nervous system, some authors have tested the hypothesis of dysfunction of nervous-system development in schizophrenia; their reports suggest that an altered NCAM level may be one of biomarkers of cognitive impairment [35]. Literature data also indicate that low concentration of NCAM in serum is associated with a smaller hippocampus volume in first-episode schizophrenia patients [40].

Thus, NCAM, ICAM, and VCAM are CAMs that affect the structure of the nervous system and cause synaptic alterations in the adult brain [41]. They are important for neuroinflammatory signal transduction across the BBB and therefore may have a role in neuroinflammatory processes in schizophrenia [16]. These proteins also take part in cell migration, axon growth, peripheral axon regeneration, and synaptic plasticity [42,43]. Despite a large number studies on CAMs and their soluble forms in schizophrenia, there have been few research articles about MetS in patients with schizophrenia.

The aim of the study was investigation of CAMs in blood serum of schizophrenia patients with MetS.

## 2. Materials and Methods

### 2.1. The Study Population and Sample Collection

The study complied with the Declaration of Helsinki (1975, revised in Fortaleza, Brazil, 2013). The Bioethical Committee of the Mental Health Research Institute of Tomsk National Research Medical Center (the Russian Academy of Sciences) approved the study protocol (decision No. 187, approval on 24 April 2018). All patients had an opportunity to familiarize themselves with the purpose and objectives of the study and received answers to their questions. Then, all participants provided written informed consent. After obtaining informed consent, we recruited 211 patients with schizophrenia being treated at various clinics in Siberia (Russian Federation): the Mental Health Research Institute of Tomsk National Research Medical Center, Tomsk Clinical Psychiatric Hospital, Kemerovo Regional Clinical Psychiatric Hospital, and N.N. Solodnikova Clinical Psychiatric Hospital of Omsk. 

The main inclusion criteria were (i) the paranoid type of schizophrenia diagnosed—consistently with ICD-10 (International Classification of Diseases, 10th revision)—according to a structured clinical interview (Structured Clinical Interview for the DSM), (ii) age 18–65 years, (iii) the patient’s informed consent, and (iv) confirmed absence of pronounced organic pathology or unstable somatic disorders. The Positive and Negative Syndrome Scale (PANSS) for schizophrenia was used to assess symptom severity [44]. This questionnaire includes three subscales that allow to separately evaluate positive, negative, and general psychopathological symptoms and to calculate the total score.

Baseline antipsychotic treatment and concomitant therapies (drugs, doses administered, and duration of current medication use) were assessed, as were previous antipsychotic and concomitant somatic therapies during the preceding 6 months. We employed the chlorpromazine equivalent (CPZeq) daily dose to standardize the dose, efficacy, and adverse effects of antipsychotics [45]. 

The diagnosis of MetS was made according to the criteria of the International Diabetes Federation (IDF, 2005) [46]; they included a definition of abdominal obesity (waist circumference greater than 94 cm in males or greater than 80 cm in females) and the presence of any two of the following four signs:

1. The level of triglycerides above 1.7 mmol/L or an ongoing lipid-lowering therapy.
2. The level of high-density lipoprotein cholesterol of less than 1.03 mmol/L in males or less than 1.29 mmol/L in females.
3. Blood pressure higher than or equal to 130/85 mm Hg or the use of antihypertensive medication.
4. The level of glucose in blood serum higher than or equal to 5.6 mmol/L or previously diagnosed type 2 diabetes mellitus.

Blood samples were collected after a 12-h overnight fast in the first days of admission to a hospital before antipsychotic-drug ingestion and were centrifuged for 30 min at 2000× *g* and 4 °C to isolate serum. The serum samples were stored at −80 °C until analysis. 

### 2.2. Laboratory Metrics

Parameters characterizing MetS, including concentrations of glucose, triglycerides, and high-density lipoprotein cholesterol, were determined in serum using Cormay kits (Lomianki, Poland) on a biochemical analyzer. Concentrations of sCAMs (sICAM-1, sNCAM, and sVCAM-1) were measured by means of Magpix and Luminex 200 multiplex analyzers (Luminex, Austin, TX, USA) and xMAP Technology at the Medical Genomics core facility of Tomsk National Research Medical Center. Panel HNDG3MAG-36K by MILLIPLEX® MAP (Merck, Darmstadt, Germany) was used to quantify the CAMs. The data were processed in the Luminex xPONENT software, with subsequent export of the results to the MILLIPLEX® Analyst 5.1 software.

### 2.3. Statistical Analysis

Data analysis was carried out using the SPSS Statistics software (version 23) for Windows. Data were checked for normality of distribution by the Shapiro–Wilk test. The significance of differences was determined by the Mann–Whitney *U* test for independent samples with a non-normal distribution along with the calculation of the median and quartiles (Me [Q1; Q3]). For normally distributed data, the results are presented as the mean and standard deviation (SD) together with significance of differences according to Student’s *t* test. The Chi-square test was applied to categorical variables. Bonferroni’s correction was used for multiple comparisons. Spearman’s correlation analysis was carried out to assess associations among the investigated parameters. Multiple regression analysis was performed to estimate the joint effect of different variables on a CAM’s level. Data were assumed to be statistically significant at *p*-values less than 0.05.

## 3. Results

### 3.1. Sociodemographic and Clinical Characteristics of the Subjects 

The main sociodemographic and clinical characteristics of the enrolled patients are shown in Table 1. 

Patients with schizophrenia were subdivided into two groups depending on the presence of MetS: a group with MetS (82 patients, 38.86%) and a control group (without MetS; 129 patients or 61.14%). Patients with MetS had statistically significantly older age, longer duration of schizophrenia, older onset age of schizophrenia, a higher BMI, and greater waist circumference. Most patients (over 90%) in the MetS group were obese or overweight, while patients in the control group had either normal weight or overweight (~50% each). The sex distribution was almost the same between the two groups. The PANSS score and CPZeq did not differ significantly between the two groups.

### 3.2. Cell Adhesion Molecules

Patients with MetS showed a significantly higher serum concentration of sICAM-1 as compared with patients without MetS (Table 2). Additionally, the level of sVCAM-1 was significantly lower in the MetS group than in patients without MetS. The concentration of NCAM in the serum did not differ significantly between the two groups.

Next, the concentrations of CAMs were collated with the patients’ weight (considered normal at BMI < 25, overweight or obesity at BMI ≥ 25; Table 3).

The serum level of sICAM-1 was found to be significantly higher in obese and overweight patients than in patients with a normal BMI.

Spearman’s correlation analysis was carried out to assess relations between the studied quantitative parameters in the control group (Table 4) and in the MetS group (Table 5). Plots of correlation between CAMs, age, duration of schizophrenia, and BMI among patients with or without MetS are presented in the Supplementary File (Figures S1 and S2).

sNCAM manifested a weak negative correlation with age, whereas sICAM-1 weakly positively correlated with the BMI in the group of patients without MetS. CAMs correlated with one another (a positive correlation on average).

The correlation analysis revealed that associations were weaker in the group of patients with MetS. For instance, there were no significant associations of CAMs with age, duration of illness, or BMI among patients with MetS. A significant correlation between sNCAM and sICAM-1 levels, which was found in patients with schizophrenia without MetS, was absent in the MetS main group, and correlations between other CAMs were weaker.

We then performed a multiple regression analysis, considering CAMs dependent variables while regarding age, duration of schizophrenia, and BMI as independent variables. We found no significant associations among these parameters (Table S1). When the BMI was replaced by MetS, only sVCAM-1 maintained an independent negative correlation with MetS (*p* = 0.034; Table 6).

## 4. Discussion

CAMs are responsible for leukocyte trafficking and may serve as a link between peripheral inflammation and CNS neuroinflammation in patients with schizophrenia by mediating signaling across the BBB and by promoting immune responses. CAMs are among the most common proteins in the nervous system and take part in synaptic plasticity and functioning [43,47,48]. We hypothesized that CAMs are dysregulated in MetS in schizophrenia patients and for this purpose measured serum levels of vascular, intracellular, and neural CAMs in schizophrenia patients with MetS as compared to those without.

First, we noticed a higher concentration of sICAM-1 in the serum of schizophrenia patients with MetS in comparison to those without MetS. An increased level of sICAM-1 was also found in obese and overweight patients with schizophrenia. In the CNS, ICAM-1 is expressed in microglial cells and astrocytes and in endothelial cells of white and gray matter of the human forebrain [21]. A number of studies indicate elevated levels of sICAM-1 in schizophrenia [16,49,50] and bipolar disorders [51,52], whereas some old articles show lower levels in schizophrenia [53,54]. We have demonstrated in some of our previous papers that MetS in schizophrenia is associated with upregulation of inflammatory factors (apolipoproteins and cytokines) [13,55]. This finding can be interpreted as the presence of an inflammatory component in the mechanism of MetS onset in individuals with schizophrenia. ICAM may play a specific role in the extension of peripheral inflammatory responses to the CNS by promoting leukocyte penetration through the BBB [56]. Cytokines such as tumor necrosis factor (TNF) regulate the permeability of the BBB to leukocytes—among other phenomena—by promoting the expression of ICAM in the endothelial cells that constitute the BBB [56,57]. It can be supposed that ICAM-1 levels are associated with hyperpermeability or hypopermeability of the BBB and thus affect the connection between glial cells and the peripheral immune system. These aberrations may not only follow the onset of MetS but also precede it, for example, when neuroinflammation occurs in behavior-modulating neuronal structures such as the dorsal diencephalic conduction system [58,59]. Intervention studies on experimental animals are needed to explore this topic further, as done previously, for example, for mRNA expression of NCAM, ICAM, and VCAM in the hippocampus of mice with streptozotocin/nicotinamide-induced diabetes [41]. There are also data on widespread expression of sICAM-1 in vessels [60] and on its participation in atherosclerosis and metabolic diseases [61,62], in which elevated levels are observed, meaning a cardiometabolic risk. Literature data suggest that insulin resistance and MetS were associated with sICAM-1 levels in Taiwanese [63].

Second, we found a statistically significant decrease in the level of sVCAM-1 in the serum of patients with schizophrenia with metabolic syndrome compared with patients without MetS in our study of Siberian White patients with schizophrenia. sVCAM-1 appears to be associated with obesity in Pima Native Americans with type 2 diabetes [64], while no such association was found in other studies in mentally healthy White individuals with diabetes [65,66]. The possible contribution of ethnicity, schizophrenia, and its treatment to the results should be explored in future studies.

According to correlation and multiple regression analysis, we found a negative relationship between the presence of MetC and the level of sVCAM-1. No significant relationships were found between age, disease duration, BMI, and CAMs. Probably, the lack of influence of age, disease duration, and BMI on CAM is due to the fact that the mechanisms of CAMs alterations in MetS are more related to other factors, such as antipsychotic therapy, which patients with schizophrenia are forced to take for a long time.

Serum NCAM levels did not differ between patients with and without metabolic syndrome. According to the literature data, the level of sNCAM is reduced in patients with schizophrenia and in patients with cognitive deficits [36,40]. NCAM’s role in the development of metabolic disorders has not previously been studied, except for the study of its expression in experimental mouse models of type 2 diabetes and the association of genetic variants at NCAM locus with lipid metabolism disorders [38,67].

This is the first study known to us of the influence of MetS on peripheral CAM levels in an adequately sized population of people with schizophrenia of homogeneous ethnicity. Our pilot study does not allow us to clarify the mechanisms of changes in CAMs in metabolic syndrome in patients with schizophrenia due to several limitations. A limitation is the trans-sectional nature of our investigation and the existence of a variety of differences between groups. Moreover, the clinical syndrome of schizophrenia has a heterogeneous biological character, and the patients were treated with a variety of antipsychotics over a long period of time. For this, medication history cannot be standardized, and it is usually not known. This increases the need for intervention studies.

However, the obtained preliminary results demonstrate a violation of leukocyte/vascular interactions, which manifests itself in an increase in sICAM-1 and a decrease in sVCAM-1, which can be identified as a novel pattern of dysregulation in the combination of metabolic syndrome and schizophrenia.

## 5. Conclusions

Our findings are consistent with reported high levels of CAMs and of some cytokines in patients with MetS [8], suggesting that MetS is associated with endothelial dysfunction along with other components of inflammation. We can hypothesize that through these endothelial components of peripheral inflammatory processes, MetS induces intracerebral neuroinflammatory changes that may participate in the pathophysiology of MetS itself and of schizophrenia, but further investigation is needed to test this theory.

## Figures and Tables

**Table 1:** The main sociodemographic and clinical characteristics of schizophrenia patients with and without MetS.

Parameter		Patients with MetS *n* = 82	Patients without MetS *n* = 129	*p*-Value
Sex	Female, *n* (%)	38 (46.3%)	70 (54.3%)	0.145
	Male, *n* (%)	44 (53.7%)	59 (45.7%)	
Age, years (Me [Q1; Q3])		44 [34; 52]	33 [28; 39]	<0.001 *
Age of SCZ onset, years (Me [Q1; Q3])		26 [21; 31]	23 [19; 29]	0.002 *
Duration of disorder, years (Me [Q1; Q3])		16 [8.75; 22]	8 [4; 15]	<0.001 *
PANSS, total score (Me [Q1; Q3])		100 [85; 109]	100 [88; 111]	0.282
Total CPZeq (Me [Q1; Q3])		434.8 [225; 687.5]	450 [250; 750]	0.962
BMI (Me [Q1; Q3])		31.15 [26.9; 35.58]	24 [21.9; 28.4]	<0.001 *
Waist circumference, cm (mean ± SD)		104.82 ± 12.08	85.83 ± 13.22	<0.001 **
Normal weight (BMI < 25), *n* (%)		8 (9.2%)	74 (57.4%)	<0.001 *
Overweight or obesity (BMI > 25), *n* (%)		74 (90.8%)	55 (42.6%)	

Notes: BMI, body-mass index; CPZeq, chlorpromazine equivalents; Me [Q1; Q3], median and quartiles; MetS, metabolic syndrome; SCZ, schizophrenia; SD, standard deviation. * Significance of differences according to the Mann–Whitney *U* test. ** Significance of differences according to Student’s *t* test.

**Table 2:** Levels of CAMs in schizophrenia patients with or without MetS (Me [Q1; Q3]).

Parameter, ng/mL	Patients with MetS *n* = 82	Patients without MetS *n* = 129	*p*-Value
sICAM-1	138.97 [104.12; 174.03]	117.88 [91.64; 157.32]	0.039 *
sNCAM	235.36 [203.26; 296.58]	247.31 [212.26; 305.12]	0.279
sVCAM-1	937.09 [813.52; 1153.77]	1027.82 [905.17; 1201.98]	0.033 *

Notes: Me [Q1; Q3], median and quartiles; MetS, metabolic syndrome; sICAM-1, soluble intercellular adhesion molecule 1; sNCAM, soluble neural cell adhesion molecule; sVCAM-1, soluble vascular cell adhesion molecule 1. * Significance of differences according to the Mann–Whitney *U* test.

**Table 3:** Levels of CAMs in schizophrenia patients depending on their weight (Me [Q1; Q3]).

Parameter, ng/mL	Normal (BMI < 25) *n* = 82	Overweight and Obesity (BMI ≥ 25) *n* = 129	*p*-Value
sICAM-1	106.63 [82.16; 138.77]	138.67 [109.07; 176.09]	<0.001 *
sNCAM	246.71 [215.03; 304.72]	240.27 [210; 30.31]	0.5
sVCAM-1	1009.25 [847.58; 1160.28]	1009.39 [871.86; 1175.2]	0.958

Notes: Me [Q1; Q3], median and quartiles; MetS, metabolic syndrome; sICAM-1, soluble intercellular adhesion molecule 1; sNCAM, soluble neural cell adhesion molecule; sVCAM-1, soluble vascular cell adhesion molecule 1; BMI, body-mass index. * Significance of differences according to the Mann–Whitney *U* test.

**Table 4:** Spearman’s analysis of correlation between CAMs, age, duration of schizophrenia, and BMI among patients without MetS.

Parameter		sICAM-1	sNCAM	sVCAM-1
Age	ρ *p*-value	0.111 0.214	−0.202 * 0.022	0.090 0.309
Duration of illness	ρ *p*-value	−0.050 0.575	−0.111 0.211	0.105 0.235
BMI	ρ *p*-value	0.204 * 0.036	−0.027 0.785	0.083 0.396
sICAM-1	ρ *p*-value	1	0.354 * <0.001	0.444 * <0.001
sNCAM	ρ *p*-value	0.354 * <0.001	1	0.501 * <0.001
sVCAM-1	ρ *p*-value	0.444 * <0.001	0.501 * <0.001	1

Notes: BMI, body-mass index; sICAM-1, soluble intercellular adhesion molecule 1; sNCAM, neural cell adhesion molecule; sVCAM-1, soluble vascular cell adhesion molecule 1; ρ, Spearman’s coefficient. * Significance of a correlation (*p* < 0.05).

**Table 5:** Spearman’s analysis of correlation between CAMs, age, duration of schizophrenia, and the BMI among patients with MetS.

		sICAM-1	sNCAM	sVCAM-1
Age	ρ *p*-value	0.021 0.851	−0.110 0.327	0.088 0.438
Duration of illness	ρ *p*-value	−0.035 0.758	−0.133 0.235	−0.081 0.476
BMI	ρ *p*-value	0.015 0.900	−0.090 0.453	−0.072 0.551
sICAM-1	ρ *p*-value	1	0.170 0.126	0.364 * 0.001
sNCAM	ρ *p*-value	0.170 0.126	1	0.438 * <0.001
sVCAM-1	ρ *p*-value	0.364 * 0.001	0.438 * <0.001	1

Notes: BMI, body-mass index; sICAM-1, soluble intercellular adhesion molecule 1; sNCAM, soluble neural cell adhesion molecule; sVCAM-1, soluble vascular cell adhesion molecule 1; ρ, Spearman’s coefficient. * Significance of a correlation (*p*-value < 0.05).

**Table 6:** Determinants of CAMs’ concentrations in multiple regression analysis, with age, duration of schizophrenia, and MetS as independent variables.

Variable	B	*p*-Value	Adjusted R^2^
sICAM-1			
Age, years	−0.0230	0.845	−0.006
Duration of illness, years	−0.045	0.675	
MetS	0.091	0.223	
sNCAM			
Age, years	−0.112	0.275	0.001
Duration of illness, years	0.002	0.984	
MetS	−0.028	0.701	
sVCAM-1			
Age, years	0.148	0.149	0.013
Duration of illness, years	−0.081	0.423	
MetS	−0.157	0.034 *	

Notes: MetS, metabolic syndrome; sICAM-1, soluble intercellular adhesion molecule 1; sNCAM, soluble neural cell adhesion molecule; sVCAM-1, soluble vascular cell adhesion molecule 1; * Significance of a relation (*p*-value < 0.05).

## Data Availability

The datasets are available on reasonable request to Svetlana A. Ivanova (ivanovaniipz@gmail.com), after approval from the Board of Directors of the Mental Health Research Institute, in accordance with local guidelines and regulations.

## Supplementary Materials

The following supporting information can be downloaded at: https://www.mdpi.com/article/10.3390/metabo13030376/s1, Figure S1: The correlation between cell adhesion molecules, age, duration of schizophrenia and BMI in patients without metabolic syndrome. Figure S2: The correlation between cell adhesion molecules, age, duration of schizophrenia and BMI in patients with metabolic syndrome. Table S1: Determinants of CAMs concentrations in multiple regression analysis with age, duration of illness and BMI as an independent variable.
