# Supplementary material for: Cell Adhesion Molecules in Schizophrenia Patients with Metabolic Syndrome
Source: Metabolites. 2023 Mar 2;13(3):376. doi: 10.3390/metabo13030376 (PMC10058418; doi:10.3390/metabo13030376)
Supplement: Supplementary file 1 [file metabolites-13-00376-s001.zip › metabolites-2191661-supplementary.pdf]

Table S1. Determinants of CAMs concentrations in multiple regression analysis with age, duration of illness and BMI as an independent variable.

Variable	B	p-Value	adjusted R^2
sICAM-1			
Age, years	-0.023	0.836	-0.016
Duration of illness, years	-0.012	0.912	
BMI,kg/m2	0.023	0.769	
NCAM			
Age, years	-0.140	0.197	0.008
Duration of illness, years	0.005	0.964	
BMI,kg/m2	-0.062	0.426	
sVCAM-1			
Age, years	0.076	0.488	-0.012
Duration of illness, years	-0.054	0.636	
BMI, kg/m2	-0.045	0.574	

Notes: BMI – body mass index; sICAM-1 – soluble intercellular adhesion molecule-1; sNCAM – soluble neural cell adhesion molecule; sVCAM-1 – soluble vascular cell adhesion molecule-1;

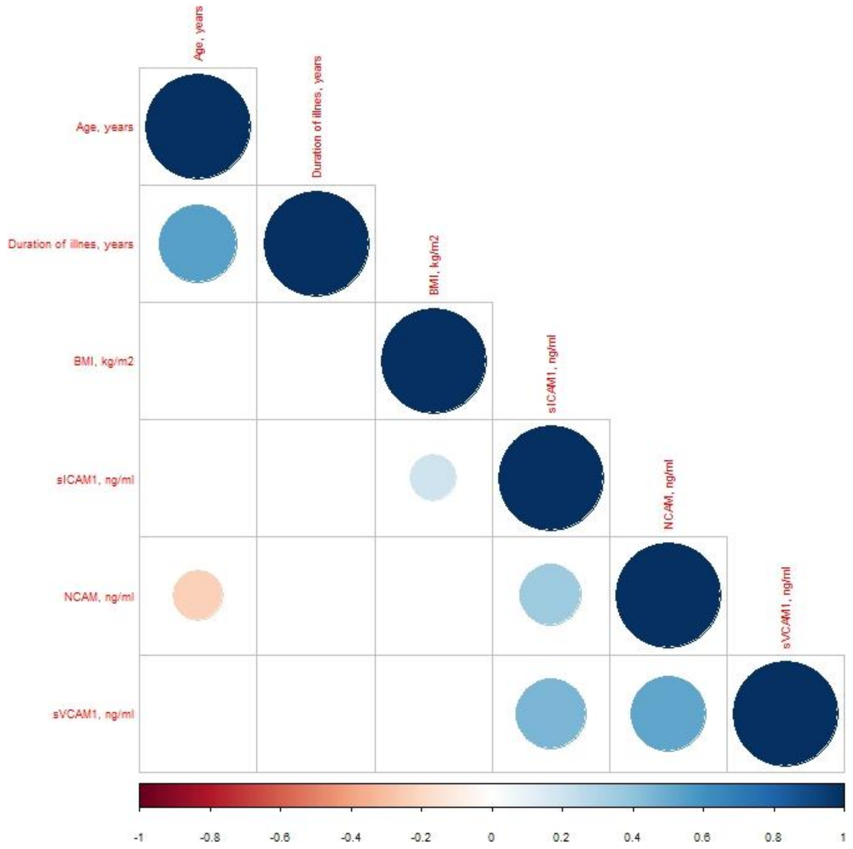

Figure S1. The correlation between cell adhesion molecules, age, duration of schizophrenia and BMI in patients without metabolic syndrome.

Note: The circles in the figure reflect only statistically significant correlations. Red and blue circles mean negative and positive correlations, respectively; the size and color intensity of circles are proportional to the correlation coefficient; in the bottom, the legend of color intensity shows the rate of correlations and the corresponding relations

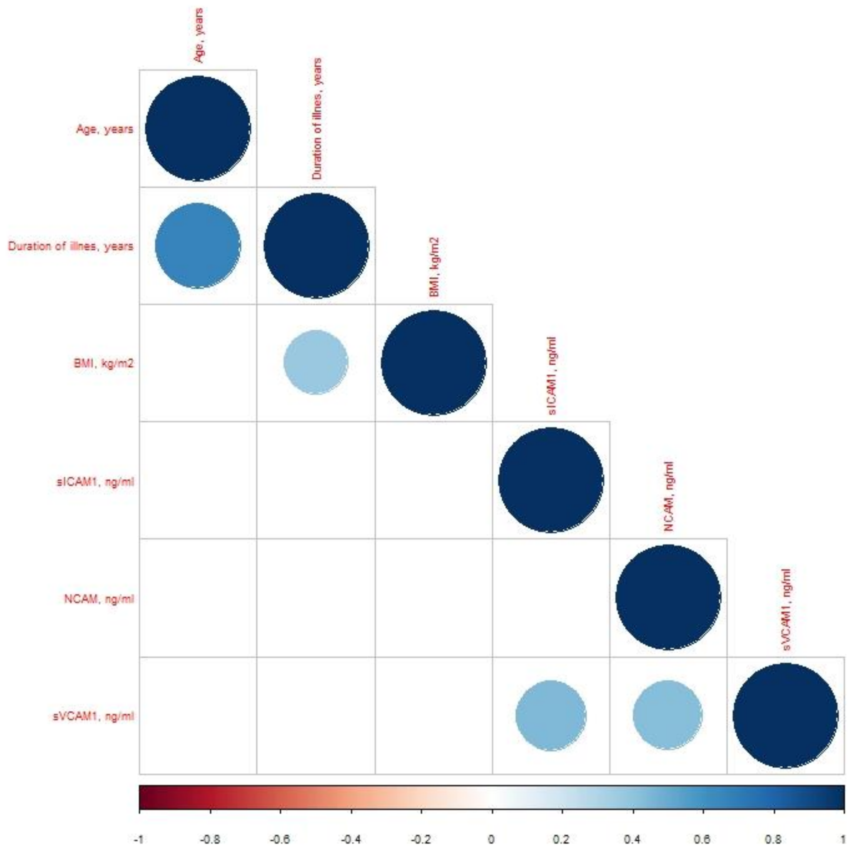

Figure S2. The correlation between cell adhesion molecules, age, duration of schizophrenia and BMI in patients with metabolic syndrome.

Note: The circles in the figure reflect only statistically significant correlations. Red and blue circles mean negative and positive correlations, respectively; the size and color intensity of circles are proportional to the correlation coefficient; in the bottom, the legend of color intensity shows the rate of correlations and the corresponding relations
